# Long-Term Effectiveness of Hepatitis B Vaccination in the Protection of Healthcare Students in Highly Developed Countries: A Systematic Review and Meta-Analysis

**DOI:** 10.3390/vaccines10111841

**Published:** 2022-10-30

**Authors:** Alborz Rahmani, Alfredo Montecucco, Bruno Kusznir Vitturi, Nicoletta Debarbieri, Guglielmo Dini, Paolo Durando

**Affiliations:** 1 Department of Health Sciences, University of Genoa, 16132 Genoa, Italy; 2 Occupational Medicine Unit, IRCCS Ospedale Policlinico San Martino, 16132 Genoa, Italy

**Keywords:** hepatitis B, HBV vaccination, healthcare students, humoral immunity, immunological memory, immune protection, occupational health, global health

## Abstract

Hepatitis B virus represents an important global health problem. In highly developed countries, mass vaccination campaigns of newborns in recent decades have drastically reduced the proportion of carriers. However, workers exposed to blood and body fluids, including healthcare students, can be at risk of exposure. In order to assess the proportion of susceptible individuals in the specific population of healthcare students in highly developed countries, a systematic review and meta-analysis was performed to summarize the evidence on the persistence of humoral immune protection induced by the primary cycle of hepatitis B vaccination, as well as the proportion of true non-responders. Forty-six studies were included in the final analysis (52,749 participants). Overall, the seroprotection prevalence at the pre-exposure assessment was equal to 73.8% (95% CI 69.1–78.0); the prevalence of anamnestic response following the administration of a challenge dose was 90.9% (95% CI 87.7–93.3), demonstrating a high proportion of persistence of vaccination-induced immunity. Among those without evidence of anamnestic response, 5.0% (95% CI 2.1–11.5) were non-responders following the completion of a secondary immunization cycle. These findings demonstrate that the majority of healthcare students vaccinated with the complete HBV primary cycle maintain an effective humoral immunity against this pathogen for over two decades.

## 1. Introduction

Hepatitis B virus (HBV) represents a common cause of viral hepatitis, with an estimated 250 million people living with chronic HBV infection globally, causing nearly 900,000 deaths every year [1]. This viral pathogen spreads through contact with contaminated body fluids and blood. Recent data from the European Centre for Disease Prevention and Control have shown that although most cases in Europe are transmitted by the sexual route and mother-to-child route, occupationally acquired infection due to needle-stick injuries and other professional exposures comprise more than 2% of cases in this region [2]. Indeed, there is still a risk for occupationally acquired hepatitis B among healthcare workers (HCWs), including healthcare students, largely due to exposures to patients with chronic HBV infection, with the estimated attributable fraction within this occupational group reaching 37% [3]. Estimates in high-income countries suggest that around 1% of all HCWs are infected with HBV [4], and although less than 5% of infected adults develop chronic infection, this progression not only represents a risk of complications, including liver cirrhosis and hepatocellular carcinoma, at the patient level, but also a source of transmission to others at the population level [5]. The widespread use of safe and effective hepatitis B vaccine since the 1980s, particularly in countries with a high degree of healthcare development, has considerably reduced the incidence of acute and chronic HBV infections [1,6]. Nonetheless, because the concentration of vaccine-induced neutralizing anti-HBs antibodies declines over time, the serologic testing of HCWs years after vaccination might not accurately distinguish responders from non-responders. For this reason, the cornerstone of the primary prevention of healthcare workers and students vaccinated with the first three-dose immunization cycle, enacted by the occupational health services in many developed nations, is to assess anti-HBs results at three time points: prior to exposure, all subjects are checked for the serologic correlate of protection (HBsAb titer ≥ 10 mIU/mL); to those who present a lower titer, a fourth booster dose is administered to challenge the immunological memory to recall the anamnestic response, with testing performed after a month; if, despite the fourth vaccine dose, the titer is still not protective, another two separate vaccine doses are administered in order to complete the secondary vaccination cycle to induce adequate immunization, and a final serologic test is performed a month following the last dose [7,8]. Indeed, only those that do not mount an appropriate immune response after the completion of the additional secondary immunization cycle (i.e., after the administration of six vaccine doses) are considered true non-responders and, therefore, susceptible to infection.

To date, however, the significance of the decline in vaccine-induced circulating antibodies, with respect to the persistence of long-term immunological memory, and the proportion of true non-responders, has not yet been clearly defined. This information is particularly useful for the fitness for work evaluation of healthcare students and trainees, a group of subjects that can be exposed to this pathogen many years after receiving the primary vaccination, and, in addition, could be at higher risk of needle-stick and sharp injuries due to the lack of experience [9,10]. Therefore, the current systematic review and meta-analysis aims to summarize the available evidence in the literature on the persistence of seroprotection induced by the three-dose primary cycle of hepatitis B vaccination among healthcare students and trainees, as well as the proportion of true non-responders in this category, in order to inform occupational health professionals and policy makers with up-to-date scientific information and key elements that ought to be taken into consideration in the workers’ health evaluation and risk assessments.

## 2. Materials and Methods

The current systematic review of the literature and meta-analysis is reported according to the Preferred Reporting Items for Systematic reviews and Meta-Analyses (PRISMA) guidelines [11].

An extensive search strategy was designed in order to retrieve all research articles reporting the prevalence of immune protection against hepatitis B virus in vaccinated healthcare students, published from 1 January 2000 to 30 November 2021, in English and Italian language, through systematic searches of major scientific databases, including PubMed/MEDLINE, Scopus, Web of Science, ProQuest, and ScienceDirect, using the UNO per TUTTO platform). Each source was last searched or consulted on 30 November 2021. In addition, a manual screening of relevant references of the included studies was performed to obtain additional studies. Studies were eligible if they met the following PICO inclusion criteria: P (population): healthcare students and medical residents; if healthcare workers were included in the study population, a 50% + 1 majority of students was required as an inclusion criterion; I (intervention): anti-HBs antibody serologic testing; C (comparator): no comparisons; O (outcome): persistence of serologic immunity induced by vaccination following: (i) anti-HBV vaccination with a three-dose primary cycle, (ii) the administration of a single booster dose in those without evidence of seroprotection, and (iii) the completion of additional two vaccine doses in those without evidence of anamnestic response. Review articles, modelling studies, case series, and case reports were excluded. When it was not possible to make a decision on a study’s inclusion or exclusion based on the title and/or abstract, the full text of the study was examined. All prevalence studies were included in the current study. A time filter was applied to include studies published since 2000, in order to better gather recent data from students who were vaccinated after the implementation of HBV vaccination policies in the 1990s. Only studies from countries with a Human Development Index (HDI) of “very high”, meaning with a value equal or higher than 0.800, were included [12], in consideration of comparable vaccination policies, higher standards of healthcare services, and a lower prevalence of HBsAg positivity in the general population, with the aim of reducing confounders. Further details of the search strategy can be found in Supplementary File S1.

The literature search was performed by two researchers independently (AR and GD). In case of disagreement, consensus was reached through discussion and consultation; in case consensus could not be reached, a third researcher (AM) acted as a final referee. The relevant information was extracted from each included article by two researchers independently (AR and BKV). For data extraction, an ad hoc Microsoft Excel (version 2203) spreadsheet was designed and utilized. From each eligible study, the following variables were extracted in the dataset: name of first author, year of publication, country, field of study, sample size, average age, gender ratio, foreigner ratio, proportion of previous first vaccination cycle, time since primary cycle, proportion of first vaccination cycle in infancy or adolescence, prevalence of seroprotection at first screening, prevalence of anamnestic response following a challenge dose, and prevalence of non-response after second cycle completion. A meta-analysis for each outcome (seroprotection after primary vaccination cycle, anamnestic response after a challenge dose, non-responders after a complete second vaccination cycle), by means of effect size (ES), was computed by pooling together the various prevalence.

The quality assessment of included studies was performed independently by two authors (AR and BKV) using the Joanna Briggs Institute Critical Appraisal Checklist tool for prevalence studies included in this review. A third author (AM) was involved to resolve disagreements regarding the quality grading.

### 2.1. Data Analysis

For every study included, we calculated the prevalence of serologic correlate of protection, anamnestic response, and non-response. The random-effects model was applied to estimate a pooled effect size. Forest plots were produced to represent all studies based on the effect size and 95% confidence interval (CI). The heterogeneity between studies was assessed using the I^2^ statistic, with a value higher than 50% considered as substantial heterogeneity [13]. Sensitivity analyses were performed by excluding individual studies from the meta-analysis in order to assess the robustness of the results. Potential publication bias was investigated visually inspecting the asymmetry of the funnel plot, and, if present, the Duval and Tweedie’s trim-and-fill analysis and the Egger’s regression test were performed [14,15]. When at least ten studies presented a specific covariate, we performed a weighted meta-regression with a random-effects model to assess the effect of moderators on the pooled effect size. A *p* < 0.05 was considered statistically significant. All statistical analyses were performed using Prometa (version 3.0) software.

### 2.2. Registration and Protocol

This review was not registered. The review protocol is available from the corresponding author on reasonable request.

## 3. Results

### Systematic Review

**Figure 1:**
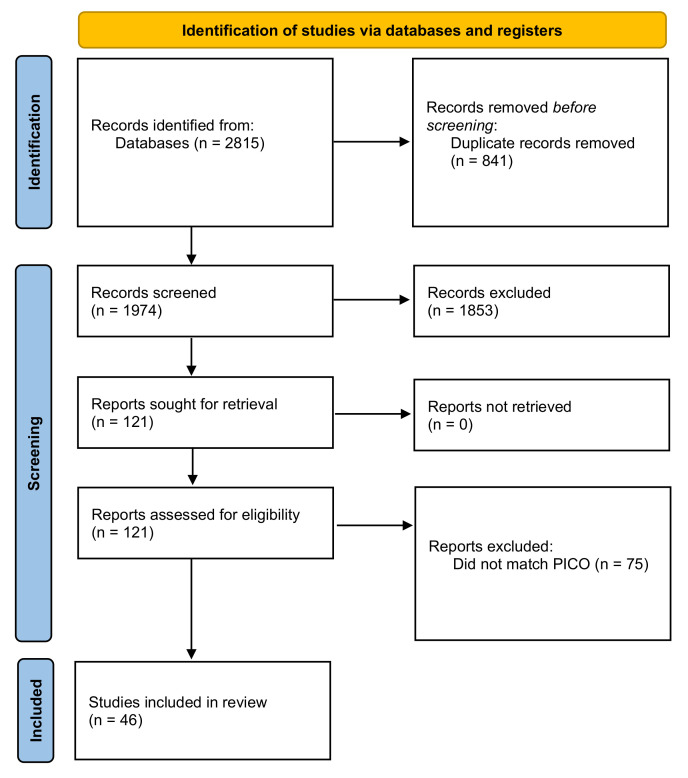
Study selection [11].

The initial systematic search resulted in a pool of 2815 potentially relevant articles. After deleting duplicates, we obtained a set of 1974 unique items. Screening titles and/or abstracts led to the exclusion of 1853 items. A pool of articles was sought for retrieval and were evaluated in the full text. After reviewing the eligibility criteria, 46 articles were included in the final qualitative and quantitative analysis (Figure 1).

Of the 46 included articles, 22 were performed in Italy, 4 in Japan, 3 in the United States and Saudi Arabia, 2 in Germany, and 1 in Australia, Cyprus, Greece, Hong Kong–China (SAR), Israel, Malaysia, the Netherlands, South Korea, Spain, Switzerland, Turkey, and the United Arab Emirates, respectively. The majority of the articles (*n* = 39) were published after 2010. Concerning the field of study of the included samples, 26 studies included overall healthcare students, 18 studies included medical students and trainees, whereas only 2 studies focused on nursing students. The critical appraisal of the methodological quality of the included studies is reported in Supplementary File S2.

The sample sizes ranged from 100 to 10,294 subjects, with a total of 52,749 participants. Between studies, the mean age ranged from 19.0 to 29.9 years; the female gender ratio ranged from 33.7% to 79.4%; the prevalence of the completed primary cycle of HBV vaccination ranged from 40.3 to 100.0%; and the time since primary cycle completion went from 0.08 to 21.4 years. The main characteristics of the included studies are presented in Table 1.

**Figure 2:**
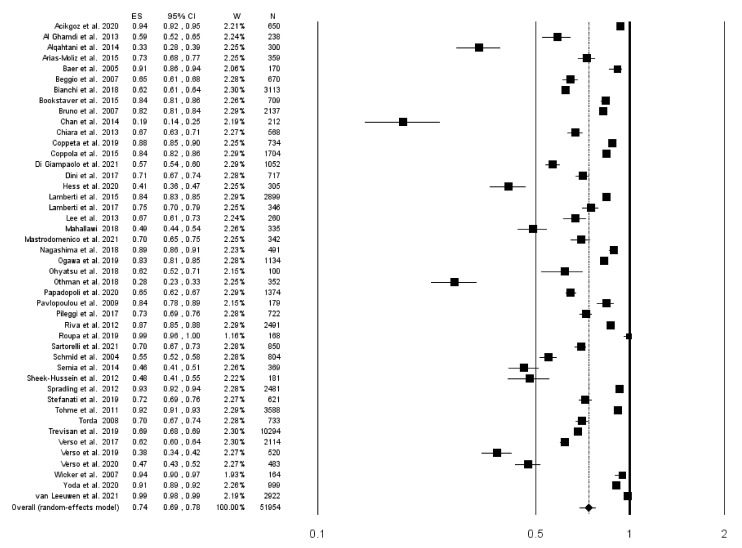
Forest plot of the prevalence of subjects with a serological correlate of protection (anti-HBs antibodies ≥10 mUI/mL).

**Figure 3:**
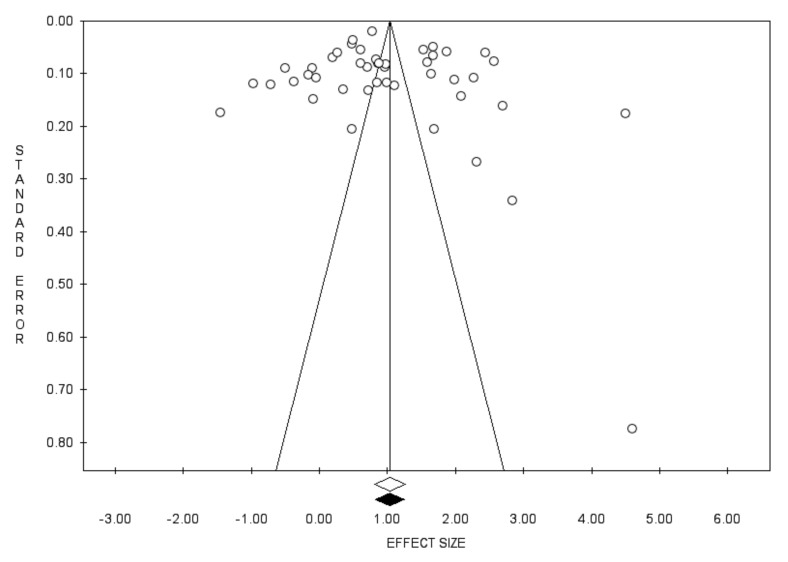
Funnel plot for the prevalence of seroprotection.

Forty-five studies reported data on the prevalence of seroprotection in the sample. Pooling the results from these studies, including 51,954 subjects, an overall prevalence of 73.8% was found (95% CI 69.1–78.0; *I*^2^ = 99.1%), with no evidence of publication bias (Figure 2 and Figure 3).

**Figure 4:**
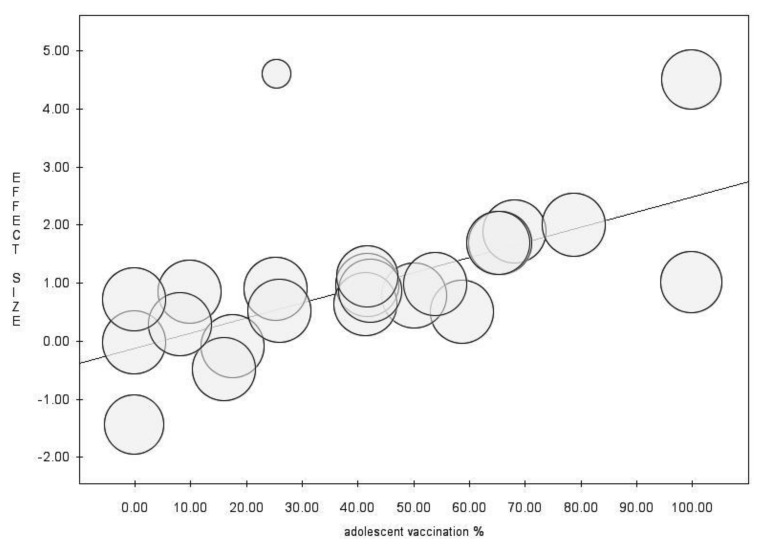
Meta-regression analysis for proportion of vaccination during adolescence, showing positive association with the proportion of seroprotected individuals.

**Figure 5:**
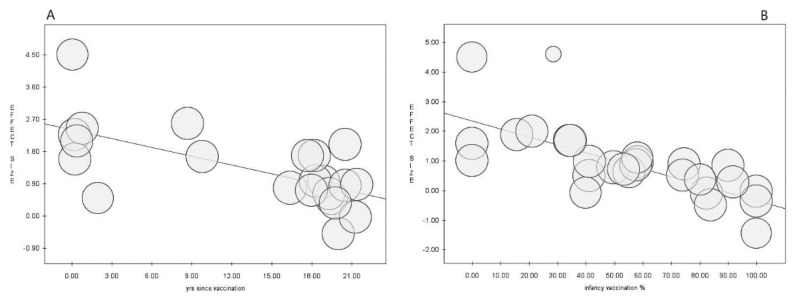
Meta-regression analysis for time (in years) since primary cycle vaccination (**A**) and proportion of vaccination during infancy (**B**), showing negative associations with the proportion of seroprotected individuals.

In the meta-regression analysis, the proportion of students vaccinated during adolescence (intercept = −0.12, slope = 0.03, *p* = 0.001) resulted in a moderator with a positive association with the prevalence of the correlate of protection (Figure 4), whereas the time past following primary immunization (intercept = 2.41, slope = −0.08, *p* = 0.001) and the proportion of students vaccinated during infancy (intercept = 2.35, slope = −0.03, *p* = 0.000) showed negative associations (Figure 5).

**Figure 6:**
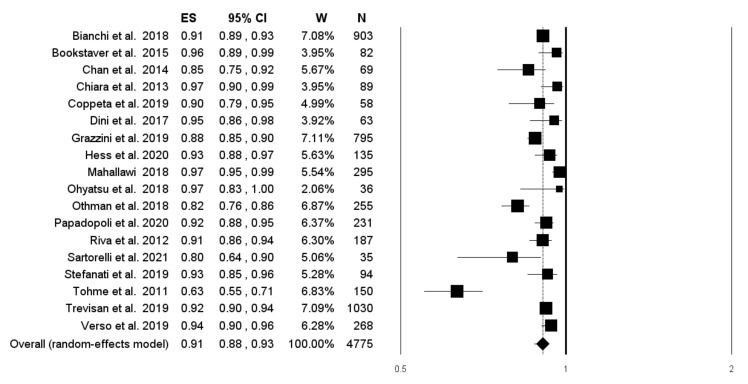
Forest plot of the prevalence of subjects showing an anamnestic response following challenge dose.

**Figure 7:**
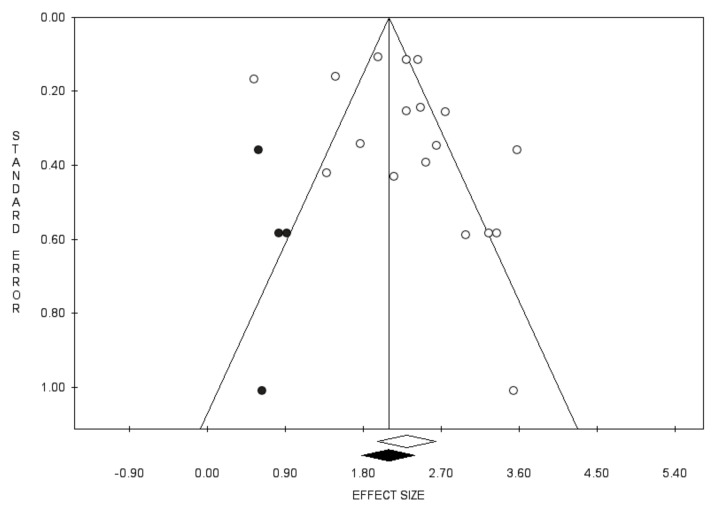
Funnel plot for the prevalence of anamnestic response following challenge dose.

Concerning the anamnestic response rate following the administration of a challenge dose, pooling the results from 18 studies, including 4775 subjects, we found a proportion of 90.9% (95% CI 87.7–93.3; *I*^2^ = 89.3%) (Figure 6), but with the presence of publication bias, identified by the visual inspection of the funnel plot, the Duval and Tweedie’s trim-and-fill analysis (Figure 7), however, with insignificant Egger’s linear regression test (intercept = 1.50, t = 1.12, *p* = 0.281).

Vaccination at infancy, adolescence, or time passed since primary immunization did not show any significant moderating effect on this outcome.

**Figure 8:**
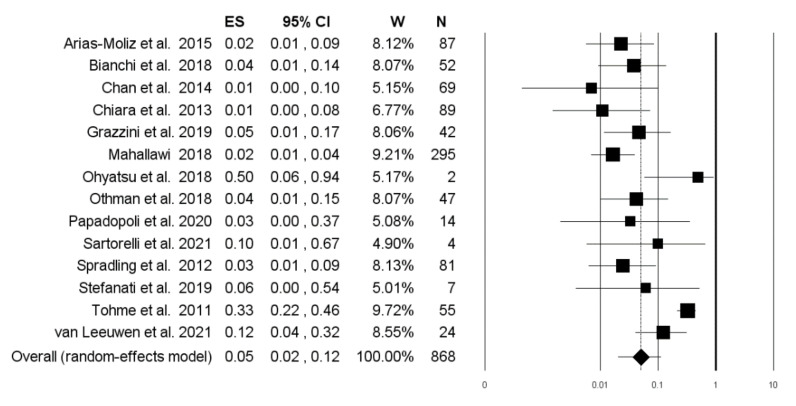
Forest plot of the prevalence of subjects not responding to the completion (fifth and sixth dose) of the second vaccination cycle.

**Figure 9:**
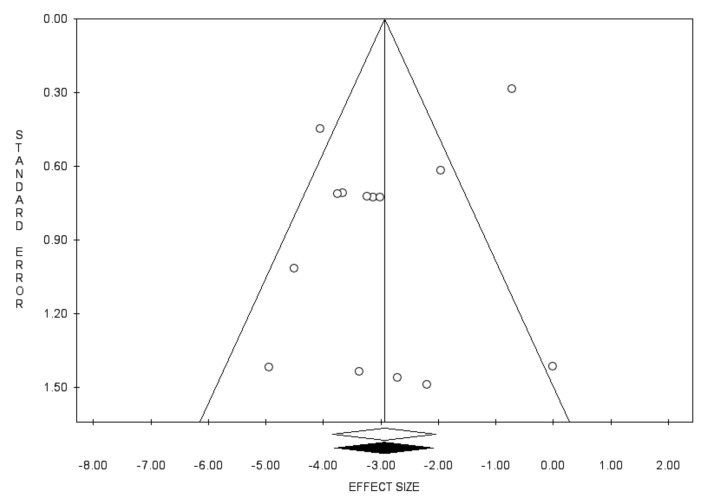
Funnel plot for the prevalence of non-response rate after the completion (fifth and sixth dose) of the second vaccination cycle.

Concerning the non-response rate following the administration of two further doses in subjects that did not show an anamnestic response, pooling the results from 14 studies, including 868 subjects, we found a proportion of 5.0% (95% CI 2.1–11.5; *I*^2^ = 81.1%), with no evidence of publication bias (Figure 8 and Figure 9).

**Figure 10:**
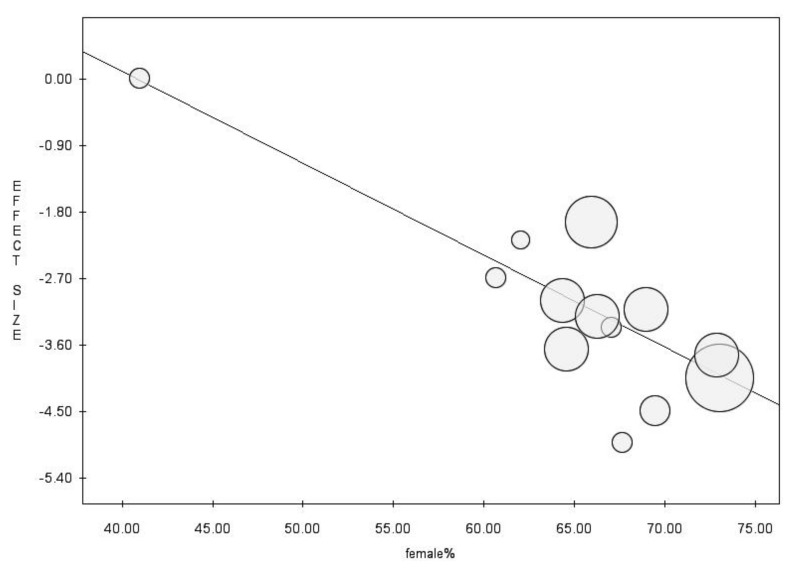
Meta-regression analysis for proportion of female gender, showing negative associations with the proportion of non-responding individuals.

At the meta-regression analysis, female gender (intercept = 5.07, slope = −0.12, *p* = 0.002) showed a negative association with the proportion of non-responders (Figure 10).

## 4. Discussion and Conclusions

To the Authors’ knowledge, this systematic review and meta-analysis is the first to specifically study and summarize the long-term protection induced by hepatitis B vaccination in healthcare students. The findings of the present study demonstrate that the vast majority of students and resident doctors vaccinated with a complete anti-HBV primary immunization cycle maintain an effective humoral immunity against this pathogen decades after vaccination. Indeed, three out of four students showed a protective HBsAb titer (≥10 mIU/mL) at the first serological assessment performed at the medical visit prior to occupational exposure. Furthermore, among those that did not show the correlate of protection, over 90% demonstrated an anamnestic response after receiving a challenge dose. The high response rate confirms the evidence of prior established immunological memory, which could have been successfully deployed following a new exposure to the wild virus in vaccinated subjects. This could be explained by the fact that waning immunity detected in peripheral blood, especially of the humoral branch, is not a completely reliable indicator of effective reduction in immunity. Indeed, only a small fraction of lymphocytes (less than 10%) is constantly circulating in the bloodstream, whereas the rest are deposited in tissues and organs [62]; this is particularly important considering that memory B cells and plasma cells, prime actors in secondary immune responses to infections (or antigen exposures), are mostly housed in bone marrow and lymphoid organs [63]. This type of immune response is particularly effective against pathogens that present long incubation periods, such as HBV; immunological memory has enough time to mount a decisive response against the infection [64]. Interestingly, although the seroprotection at the first assessment was found to be negatively associated with the length of time since primary cycle vaccination, this association was not found with the anamnestic response, indicating a possible waning immunity effect only in the peripheral blood, with a persistence of immunity in the lymphoid organs.

When considering individuals who had not shown an anamnestic response following the challenge dose administration, and that received another two doses of vaccination in order to complete a secondary immunization cycle, it was found that only 1 in 20 did not develop a protective immune reaction, and, thus, could be considered as true non-responders and potentially susceptible to the occupational risk of this bloodborne infection. Taking into account all the findings from this study, we can estimate that in a population of healthcare students in developed countries, 2.4% might be not protected at the baseline pre-exposure visit (i.e., those with a non-protective antibody titer), with only 0.1% of true non-responders after the completion of the secondary cycle. These findings show that the vast majority of people that have undergone the full primary cycle vaccination responded in an effective way, even though the baseline peripheral blood evaluation may have shown a measurement of antibodies lower than the correlate of protection. Moreover, previous studies have also suggested that any presence of humoral immunity in the peripheral blood, often regarded as a HBsAb titer higher than 2 mIU/mL, the limit of detection of most serological tests used to assess this value, might be sufficient for immune protection against HBV infection [65].

Interestingly, the results showed a reduced association between female students and non-responders, suggesting a mediating role in the immune response to HBV vaccination due to sexual differences, that can be caused by multiple factors, mainly biological ones, including hormonal and genetic [66,67,68].

Based on these results, occupational physicians could prioritize the HBV risk assessment and management of individual healthcare workers and students, taking into consideration the health and immune status of the subject, the specific tasks and procedures, as well as the prevalence of HBV patients in the community and in the specific workplace. Indeed, thanks to the mass vaccination campaigns enacted in many developed countries in the past decades, the prevalence of the contagious reservoir of HBsAg-positive has dramatically reduced in these countries, reaching levels of around 1% in regions such as Europe. However, specific occupational settings, such as healthcare systems, and also prisons and homeless shelters, and populations such as injection drug users and migrants from endemic areas, represent non negligible pockets of potential infective sources [69].

Although public health’s objectives of reducing the transmission of the disease, and the prevalence of the infected reservoir not requiring the systematic demonstration of acquired immunity after primary cycle immunization thanks to the highly effective vaccines available, from an occupational health perspective of reducing the risk and protecting all individual exposed workers, and in accordance to Italian national regulations, evaluations performed before exposing workers and students to potential bloodborne risks require the assessment of the HBsAb titer, with a recommendation to proceed to further vaccinations if less than the 10 mIU/mL cut-off is measured [8,70]. The present results support preventive strategies for healthy healthcare students based on a more tailored and personalized risk assessment, considering that most of these individuals, if not all, have been already vaccinated with a complete primary vaccination cycle during childhood or adolescence, thus reaching protection against HBV, and do not generally perform high-risk procedures [71].

The findings of this study are strengthened by the comprehensive and rigorous methodological approach adopted in the literature search and study quality assessment. However, this study presents some limitations, namely the different types of vaccination used in the included studies (e.g., plasma derived and recombinant vaccines, accurate dosages, and timing of administrations), no study assessed the effectiveness among immunocompromised students (e.g., effect of corticosteroid treatment, HIV infection, treatment with biologics), and the quantitative analysis identified substantial heterogeneity, suggesting ample differences between the included study populations. Studies improving on these limitations could further expand our knowledge and widen the scope of the implications to all exposed workers and individuals.

In conclusion, the present findings show that the vast majority of vaccinated healthcare students remain protected against HBV infection decades after the primary immunization cycle. These results can inform occupational physicians, who are in the unique position to assess each individual student’s risks based on specific health susceptibilities and the presence of serological markers of immunity, with the aim of ensuring fitness for work in specific training placements and tasks, collaborating with other occupational health and safety professionals. Further research should be performed to assess whether any evidence of humoral immunity induced by vaccination, even with lower antibody concentrations than the currently accepted cutoff (i.e., values ranging between the detection limit of the serological test and the protective threshold of 10 mIU/mL), could be considered sufficient to reach and, more importantly, to maintain protection against HBV infection in occupational settings.

## Figures and Tables

**Table 1:** Characteristics of the studies included in the current meta-analysis.

Name	Year	Country	Field of Study	Sample Size	Age (y)	Female (%)	Foreigner (%)	Vaccinated with 3-Dose Primary Cycle (%)	Time Since Primary Cycle Vaccination (y)	Primary Cycle during Infancy (%)	Primary Cycle during Adolescence (%)	HBsAb ≥ 10 mIU/mL (ratio)	Anamnestic Response Following Challenge Dose (ratio)	Non-Response Following Completion of Second Cycle n (ratio)
**Acikgoz et al.** [16]	2020	Turkey	healthcare	650	19.8	66.5	NA	NA	NA	NA	NA	0.937	NA	NA
**Al Ghamdi et al.** [17]	2013	Saudi Arabia	medical	238	22.2	76.5	NA	100.0	19.8	80.3	NA	0.588	NA	NA
**Alqahtani et al.** [18]	2014	Saudi Arabia	healthcare	300	20.9	33.7	NA	52.0	NA	NA	NA	0.33	NA	NA
**Arias-Moliz et al.** [19]	2015	Spain	dental	359	20.1	72.9	NA	100.0	7.0	0.0	100.0	0.73	NA	2 (0.023)
**Baer et al.** [20]	2005	Switzerland	medical	170	26.0	61.0	19.0	85.0	NA	NA	NA	0.91	NA	NA
**Beggio et al.** [21]	2007	Italy	healthcare	670	23.9	69.3	33.0	71.2	NA	NA	NA	0.647	NA	NA
**Bianchi et al.** [22]	2018	Italy	medical (including residents)	3113	24.0	66.3	NA	100.0	NA	74.0	26.0	0.623	0.909	2 (0.038)
**Bookstaver et al.** [23]	2015	USA	healthcare	709	24.0	62.0	NA	95.5	9.8	NA	NA	0.839	0.963	2 (missing denominator)
**Bruno et al.** [24]	2007	Italy	healthcare	2137	22.9	70.8	NA	70.5	NA	NA	NA	0.823	NA	NA
**Chan et al.** [25]	2014	Hong Kong—China SAR	medical and nursing	212	19.0	67.7	NA	100.0	NA	100.0	0.0	0.189	0.855	0 (0)
**Chiara et al.** [26]	2013	Italy	medical	568	21.0	69.5	NA	100.0	18.0	53.3	0.0	0.67	0.966	1 (0.011)
**Coppeta et al.** [27]	2019	Italy	healthcare (including workers)	734	29.9	65.5	NA	100.0	20.5	21.1	78.9	0.880	0.897	NA
**Coppola et al.** [28]	2015	Italy	healthcare	1704	26.0	60.6	1.1	100.0	17.7	34.6	65.4	0.842	NA	NA
**Di Giampaolo et al.** [29]	2021	Italy	healthcare	1052	21.2	66.3	NA	100.0	NA	91.7	8.3	0.568	NA	NA
**Dini et al.** [30]	2017	Italy	healthcare	717	24.8	67.2	NA	100.0	21.4	74.6	25.4	0.707	0.952	NA
**Grazzini et al.** [31]	2019	Italy	healthcare	795	NA	64.4	0	100.0	19.6	81.6	18.4	0	0.878	2 (0.047)
**Hess et al.** [32]	2020	Israel	healthcare	305	21.9	79.4	NA	100.0	NA	100.0	NA	0.41	0.934	NA
**Lamberti et al.** [33]	2015	Italy	healthcare	2899	26.0	60.1	1.1	100.0	18.2	34.3	65.7	0.842	NA	NA
**Lamberti et al.** [34]	2017	Italy	dental	346	26.5	47.1	0.28	100.0	NA	58.1	41.9	0.751	NA	NA
**Lee et al.** [35]	2013	South Korea	medical and nursing	260	22.8	60.4	NA	NA	NA	NA	NA	0.673	NA	NA
**Mahallawi** [36]	2018	Saudi Arabia	medical	335	22.8	73.1	0	100.0	21.3	100.0	0.0	0.49	0.973	5 (0.017)
**Mastrodomenico et al.** [37]	2021	Italy	healthcare	342	26.1	63.2	NA	100.0	20.5	57.6	42.4	0.699	NA	NA
**Nagashima et al.** [38]	2018	Japan	medical and dental	491	22.7	41.1	NA	100.0	0.4	NA	NA	0.89	NA	NA
**Ogawa et al.** [39]	2019	Japan	medical and nursing	1134	20.0	57.6	0	100.0	0.27	0	0	0.829	NA	NA
**Ohyatsu et al.** [40]	2018	Japan	medical	100	21.4	41.0	NA	100.0	2.0	NA	NA	0.62	0.972	1 (0.50)
**Othman et al.** [41]	2018	Malaysia	medical	352	NA	69.0	26.8	100.0	NA	NA	NA	0.276	0.816	2 (0.042)
**Papadopoli et al.** [42]	2020	Italy	healthcare	1374	24.8	67.1	NA	100.0	19.3	55.3	41.5	0.649	0.922	0 (0)
**Pavlopoulou et al.** [43]	2009	Greece	medical and nursing	179	23.8	66.0	7.0	77.7	NA	NA	NA	0.844	NA	NA
**Pileggi et al.** [44]	2017	Italy	healthcare (including residents)	722	25.5	70.0	NA	100.0	18.3	41.3	54.0	0.726	NA	NA
**Riva et al.** [45]	2012	Italy	healthcare (including residents)	2491	21.5	70.9	NA	100.0	NA	15.8	68.3	0.867	0.909	NA
**Roupa et al.** [46]	2019	Cyprus	healthcare	168	23.6	69.0	14.9	40.5	NA	28.6	25.6	0.988	NA	NA
**Sartorelli et al.** [47]	2021	Italy	healthcare	850	24.0	62.1	4.6	98.6	NA	90.0	10.0	0.698	0.80	0 (0)
**Schmid et al.** [48]	2004	Germany	medical and dental	804	22.4	63.4	NA	58.0	NA	NA	NA	0.55	NA	NA
**Sernia et al.** [49]	2014	Italy	healthcare	369	25.4	71.0	NA	84.8	NA	NA	NA	0.46	NA	NA
**Sheek-Hussein et al.** [50]	2012	UAE	medical	181	21.2	61.0	NA	40.3	NA	40.0	NA	0.48	NA	NA
**Spradling et al.** [51]	2012	USA	healthcare	2481	23.2	64.6	14.0	100.0	8.7	NA	NA	0.929	NA	2 (0.025)
**Stefanati et al.** [52]	2019	Italy	medical (including residents)	621	24.6	60.7	NA	100.0	18.9	58.1	41.9	0.723	0.926	0 (0)
**Tohme et al.** [53]	2011	USA	healthcare	3588	NA	NA	NA	59.7	0.81	NA	NA	0.92	0.633	18 (0.327)
**Torda et al.** [54]	2008	Australia	medical	733	NA	NA	55.0	44.2	NA	NA	NA	0.705	NA	NA
**Trevisan et al.** [55]	2019	Italy	healthcare	10294	20.8	65.8	0	100.0	16.4	49.8	50.2	0.685	0.920	NA
**van Leeuwen et al.** [56]	2021	Netherlands	medical	2922	19.5	66.0	NA	100.0	0.08	0	100.0	0.989	NA	3 (0.125)
**Verso et al.** [57]	2017	Italy	medical (including residents)	2114	26.6	39.7	0.1	100.0	19.5	41.1	58.9	0.619	NA	NA
**Verso et al.** [58]	2019	Italy	nursing	520	21.9	65.2	NA	100.0	20.0	83.8	16.2	0.377	0.94	NA
**Verso et al.** [59]	2020	Italy	nursing	483	21.7	66.9	NA	100.0	NA	82.4	17.6	0.474	NA	NA
**Wicker et al.** [60]	2007	Germany	medical	164	NA	65.5	NA	100.0	NA	NA	NA	0.945	NA	NA
**Yoda et al.** [61]	2021	Japan	medical	999	20.1	67.7	NA	97.7	0.25	NA	NA	0.906	NA	NA

Abbreviation: NA, not available.

## Data Availability

The data that support the findings of this study are available on request from the corresponding author.

## Supplementary Materials

The following supporting information can be downloaded at: https://www.mdpi.com/article/10.3390/vaccines10111841/s1, Supplementary File S1: Search strategy; Supplementary File S2: Critical appraisal of prevalence studies.
