# Supplementary material for: Long-Term Effectiveness of Hepatitis B Vaccination in the Protection of Healthcare Students in Highly Developed Countries: A Systematic Review and Meta-Analysis
Source: Vaccines (Basel). 2022 Oct 30;10(11):1841. doi: 10.3390/vaccines10111841 (PMC9695994; doi:10.3390/vaccines10111841)
Supplement: Supplementary file 1 [file vaccines-10-01841-s001.zip › Supplementary File S1.pdf]

Supplementary File S1: Search strategy

**Search strategy**	**Details**
**Search query**	("hepatitis B" OR hbv) AND (vaccin* OR vaccination OR immunization) AND (immunity OR serology OR seroprevalence OR seroepidemiology OR "anti-hbs antibody") AND ("healthcare student" OR "medical student" OR "nursing student" OR "medical college" OR "medical university")
**Databases**	PubMed/MEDLINE, Scopus, Web of Science, ProQuest, BioMedCentral, ScienceDirect
**Time filter**	January 1, 2000 - November 30, 2021
**Language filter**	English and Italian
**Inclusion criteria**	P (population): healthcare students in Countries with a very high (≥ 0.800) Human Development Index (HDI), based on HDRO 2020 data*<br><br>I (intervention): anti-HBs antibody serologic testing<br><br>C (comparator): no comparisons<br><br>O (outcome): persistence of serologic immunity induced by vaccination after anti-HBV vaccination with a 3-dose primary cycle, administration of a single booster dose in those without evidence of seroprotection, completion of additional two vaccine doses in those without evidence of anamnestic response<br><br>Study type and design: primary research, studies reporting prevalence data
**Exclusion criteria**	Studies not matching the defined PICO criteria; Review articles, modelling studies, case series and case reports

*Countries with "very high human development" according to HDRO calculations based on data from UNDESA (2019a), UNESCO Institute for Statistics (2020), United Nations Statistics Division (2020b), World Bank (2020a), Barro and Lee (2018) and IMF (2020), in alphabetic order:
Andorra; Argentina; Australia; Austria; Bahamas; Bahrain; Barbados; Belarus; Belgium; Brunei Darussalam; Bulgaria; Canada; Chile; Costa Rica; Croatia; Cyprus; Czechia; Denmark; Estonia; Finland; France; Georgia; Germany; Greece; Hong Kong, China (SAR); Hungary; Iceland; Ireland; Israel; Italy; Japan; Kazakhstan; Korea (Republic of); Kuwait; Latvia; Liechtenstein; Lithuania; Luxembourg; Malaysia; Malta; Mauritius; Montenegro; Netherlands; New Zealand; Norway; Oman; Palau; Panama; Poland; Portugal; Qatar; Romania; Russian Federation; Saudi Arabia; Serbia; Singapore; Slovakia; Slovenia; Spain; Sweden; Switzerland; Turkey; United Arab Emirates; United Kingdom; United States; Uruguay
