# Supplementary material for: Long-Term Effectiveness of Hepatitis B Vaccination in the Protection of Healthcare Students in Highly Developed Countries: A Systematic Review and Meta-Analysis
Source: Vaccines (Basel). 2022 Oct 30;10(11):1841. doi: 10.3390/vaccines10111841 (PMC9695994; doi:10.3390/vaccines10111841)
Supplement: Supplementary file 1 [file vaccines-10-01841-s001.zip › Supplementary File S2.pdf]

Supplementary File S2. Critical appraisal of prevalence studies included in the present systematic review and meta-analysis.

**Study**	**Domain 1**	**Domain 2**	**Domain 3**	**Domain 4**	**Domain 5**	**Domain 6**	**Domain 7**	**Domain 8**	**Domain 9**
Acikgoz et al., 2020	Yes	Yes	Yes	Yes	Yes	Yes	Yes	Yes	Yes
Al Ghamdi et al., 2013	Yes	Yes	No	Yes	Yes	Yes	Yes	Yes	Yes
Alqahtani et al., 2014	Yes	Yes	No	Yes	Yes	Yes	Yes	Yes	Yes
Arias-Moliz et al., 2015	Yes	Unclear	Yes	Yes	Yes	Yes	Yes	Yes	Yes
Baer et al., 2005	Yes	Not reported	No	Yes	Yes	Yes	Yes	Yes	Yes
Beggio et al., 2007	Yes	Unclear	Yes	Yes	Yes	Yes	Yes	Yes	Yes
Bianchi et al., 2018	No	Yes	Yes	Yes	Yes	Yes	Yes	Yes	Yes
Bookstaver et al., 2015	Yes	Yes	Yes	Yes	Yes	Yes	Yes	Yes	Yes
Bruno et al., 2007	Yes	Unclear	Yes	Yes	Yes	Yes	Yes	Yes	Yes
Chan et al., 2014	Yes	Unclear	No	Yes	Yes	Yes	Yes	Yes	No
Chiara et al., 2013	Yes	Yes	Yes	Yes	Yes	Yes	Yes	Yes	No
Coppeta et al., 2019	No	Unclear	Yes	Yes	Yes	Yes	Yes	Yes	No
Coppola et al., 2015	Yes	Yes	Yes	Yes	Yes	Yes	Yes	Yes	Yes
Di Giampaolo et al, 2021	Yes	Yes	Yes	Yes	Yes	Yes	Yes	Yes	Yes

Dini et al., 2017	Yes	Yes	Yes	Yes	Yes	Yes	Yes	Yes	No
Grazzini et al, 2019	Yes	Yes	Yes	Yes	Yes	Yes	Yes	Yes	Yes
Hess et al., 2020	Yes	Yes	No	Yes	Yes	Yes	Yes	Yes	Yes
Lee et al., 2013	Yes	No	No	Yes	Yes	Yes	Yes	Yes	Yes
Lamberti et al., 2015	Yes	Unclear	Yes	Yes	Yes	Yes	Yes	Yes	Yes
Lamberti et al., 2017	Yes	Unclear	Yes	Yes	Yes	Yes	Yes	Yes	Yes
Mahallawi, 2018	Yes	Yes	Yes	Yes	Yes	Yes	Yes	Yes	Yes
Mastrodomenico et al., 2021	Yes	Yes	Yes	Yes	Yes	Yes	Yes	Yes	Yes
Nagashima et al., 2018	Yes	Unclear	Yes	Yes	Yes	Yes	Yes	Yes	Yes
Ogawa et al., 2019	Yes	Unclear	Yes	Yes	Yes	Yes	Yes	Yes	Yes
Ohyatsu et al., 2018	Yes	No	No	Yes	Yes	Yes	Yes	Yes	Yes
Othman et al., 2018	Yes	No	Yes	No	Yes	Yes	Yes	Yes	Yes
Papadopoli et al., 2020	Yes	Yes	Yes	Yes	Yes	Yes	Yes	Yes	Yes
Pavlopoulou et al., 2009	Yes	Unclear	No	Yes	Yes	Yes	Yes	Yes	Yes
Pileggi et al., 2017	No	Yes	Yes	Yes	Yes	Yes	Yes	Yes	Yes
Riva et al., 2012	No	Unclear	Yes	Yes	Yes	Yes	Yes	Yes	No

Roupa et al., 2019	Yes	No	No	Yes	Yes	Yes	Yes	Yes	Yes
Sartorelli et al., 2021	Yes	Yes	Yes	Yes	Yes	Yes	Yes	Yes	Yes
Schmid et al., 2004	Yes	Yes	Yes	Yes	Yes	Yes	Yes	Yes	Yes
Sernia et al., 2014	Yes	Unclear	Yes	Yes	Yes	Yes	Yes	Yes	Yes
Sheek-Hussein et al., 2012	Yes	Yes	No	Yes	Yes	Yes	Yes	Yes	Yes
Spradling et al., 2012	Yes	Unclear	Yes	Yes	Yes	Yes	Yes	Yes	Yes
Stefanati et al., 2019	No	No	Yes	Yes	Yes	Yes	Yes	Yes	No
Tohme et al., 2011	Yes	Unclear	Yes	No	Yes	Yes	Yes	Yes	No
Torda et al., 2008	Yes	No	Yes	No	Yes	Yes	Yes	Yes	Yes
Trevisan et al., 2019	Yes	Yes	Yes	Yes	Yes	Yes	Yes	Yes	Yes
van Leeuwen et al., 2021	Yes	Yes	Yes	Yes	Yes	Yes	Yes	Yes	Yes
Verso et al., 2017	No	Yes	Yes	Yes	Yes	Yes	Yes	Yes	Yes
Verso et al., 2019	Yes	Unclear	Yes	Yes	Yes	Yes	Yes	Yes	Yes
Verso et al., 2020	Yes	Unclear	Yes	Yes	Yes	Yes	Yes	Yes	Yes
Wicker et al., 2007	Yes	Yes	No	No	Yes	Yes	Yes	Yes	Yes
Yoda et al., 2021	Yes	Yes	Yes	Yes	Yes	Yes	Yes	Yes	Yes
